# Pain outside the body: defensive peripersonal space deformation in trigeminal neuralgia

**DOI:** 10.1038/s41598-017-12466-5

**Published:** 2017-10-02

**Authors:** R. J. Bufacchi, C. F. Sambo, G. Di Stefano, G. Cruccu, G. D. Iannetti

**Affiliations:** 1 Department of Neuroscience, Physiology and Pharmacology, University College London (UCL), London, WC1E 6BT, UK; 2 Centre for Mathematics and Physics in the Life Sciences and EXperimental biology (CoMPLEX), University College London, London, WC1E 6BT, UK; 3 Department of Neurology and Psychiatry, Sapienza University, Viale dell‘Università 30, 00185 Rome, Italy

## Abstract

Perception of space has been guiding effective therapeutic interventions in a number of unilateral chronic pain conditions. However little is known about how trigeminal neuralgia (TN), a condition in which trigeminal stimulation triggers paroxysmal facial pain, affects defensive peripersonal space (DPPS), the portion of space surrounding the body within which defensive responses are enhanced. Given that TN is unilateral, in TN patients the DPPS of the face might not be horizontally symmetric as in pain-free individuals, but instead larger around the affected side. We tested this *a priori* hypothesis by measuring the proximity-dependent modulation of the hand-blink reflex. Stimuli delivered to the hand ipsilateral to TN elicited a stronger blink, particularly when it was measured from the eye ipsilateral to TN and the hand was closer to the face. Geometric modelling revealed (1) that DPPS was larger on the side of space ipsilateral to TN, and (2) this asymmetry was consequent to an increased estimated potential of sensory events to cause harm when they occur ipsilaterally to TN. These observations demonstrate that neural mechanisms underlying body protection in TN are adjusted to reduce the likelihood that external events evoke the painful paroxysm typical of this condition.

## Introduction

The defensive peripersonal space (DPPS) is a portion of space surrounding the body with a crucial protective function. Whenever a potentially dangerous stimulus approaches or enters this area, the individual engages in protective actions aimed at avoiding or minimising harm^1^. The strength of such defensive responses is modulated by the estimated potential of the threat to harm the individual^2^, and the distance between the threat and the body is a crucial determinant of that potential^3,4^.

We have previously identified the DPPS surrounding the face in humans by recording the eye blink elicited by electrical stimulation of the median nerve at the wrist (hand-blink reflex, HBR). The HBR is enhanced when the hand is placed near the eye^5,6^. This enhancement results from top-down modulation of the brainstem circuits mediating the HBR^5^, possibly arising from the fronto-parietal cortical network involved in representing the peripersonal space^7,8^ and in detecting potential threats near the body^5,9–11^.

Geometrical modeling of the HBR enhancement has allowed derivation of the fine-grained spatial properties of the DPPS, which is anchored to the face and has the shape of a bubble elongated along the vertical axis^12^. The DPPS boundary (defined as the position of the first robust HBR magnitude increase at group level) is located between 20 and 40 cm from the face, and within this space HBR responses are further enhanced with proximity of the threat to the face, thus defining a high-risk area^12,13^. There are clear inter-individual differences in DPPS extension, which co-varies with trait anxiety: in highly anxious participants the boundary of the DPPS is at a greater distance from the face compared to less anxious participants^13^.

Another important factor in determining the estimated potential of a threat to do harm, and thereby alter the spatial properties of the DPPS, is the state of the somatosensory system. Environmental stimuli occurring in spatial proximity of the area of hyperalgesia surrounding an injury are bound to be considered as a stronger threat. Chronic pain patients with hyperalgesia or allodynia may be highly sensitive to environmental stimuli close to the affected area, because of their potential to evoke a painful sensation.

Trigeminal neuralgia is a unique condition characterized by paroxysmal pain attacks of abrupt onset and very short duration, typically lasting few seconds and recurring with highly variable frequency: from 1 to over 50 a day^14–18^. The pain is most often described as stabbing or electric shock-like sensations, which are characteristically evoked (*trigger manoeuvre*) by innocuous somatosensory stimuli, such as a gentle touch, a breeze or shaving, in specific areas (*trigger zones*) within the affected division of the trigeminal territory.

Recent classifications distinguish two TN phenotypes, typical and atypical (the latter presenting with concomitant continuous pain in addition to the typical paroxysmal pain) with three possible aetiological forms: *classical* TN (the cause is compression of the trigeminal root by an arterial anomalous course—most often the superior cerebellar artery), *secondary* TN (the cause is multiple sclerosis or a benign tumour at the cerebellopontine angle), and *idiopathic* TN (no cause can be found)^14–18^.

TN being a strictly unilateral condition, it is possible to hypothesize that in these patients the DPPS surrounding the face is not symmetrically distributed as in pain-free individuals^12^, but is instead larger around the affected side.

Here we tested this hypothesis by exploiting formal geometrical modelling to map the DPPS of the face in 20 patients affected by TN. This allowed us to explore the functioning of neural systems underlying danger assessment and defensive behaviours in health and disease.

## Materials and Methods

### Patients

Twenty patients (11 women) with classical or idiopathic trigeminal neuralgia (TN), aged 41–75 (*M* = 56.2, *SD* = 9.4), voluntarily participated in the study. These patients were identified as HBR responders^5,13,19,20^, out of a group of patients recruited from the pain clinic of the Department of Neurology and Psychiatry, Sapienza University of Rome (29 patients), and the Trigeminal Neuralgia Association, UK (TNA UK) (two patients). The proportion of HBR responders as part of the initial sample (63%) was consistent with that found by us in healthy participants^5,6,12,13,21^. We selected patients with the most typical presentation and aetiology of TN to minimize the possibility that different pathophysiological mechanisms gave rise to even slightly different perceptions of pain. Therefore, inclusion criteria were: (a) age of 30–80 years; (b) paroxysmal pain distributed to one or more trigeminal divisions unilaterally, (c) presence of one or more trigger zones, i.e. areas of the face which, when touched, cause an attack of pain, (d) typical TN presentation (i.e. purely paroxysmal TN), as defined by the new classification of the International Association for the Study of Pain (IASP) and the European Academy of Neurology (EAN)^15^; and (e) duration of disease over 3 months. All participants gave written informed consent to take part in the study. The study was approved by the ethics committees of Sapienza, University of Rome, and University College London. All experiments were performed in accordance with the Declaration of Helsinki, as well as local guidelines and regulations.

Patients were excluded if they had secondary TN (as demonstrated by MRI or trigeminal reflex testing^14^), or they had a history of any other neurological or psychiatric disorder. All patients were on an active phase of TN, based both on the patient’s report and the presence of trigger zones. Patients under pharmacological therapy (with oxcarbazepine or carbamazepine) were asked to suspend medication for at least 12 hours prior to their participation in the study.

### Stimulation and recording

Electrical stimuli were delivered transcutaneously to the median nerve at the wrist using a bipolar electrode. The duration of each stimulus was 200 μs, and the inter-stimulus interval (ISI) was ~30s^5^. Electromyographic (EMG) signals were recorded bilaterally from the *orbicularis oculi* muscle using two pairs of surface electrodes; the active electrode was placed over the mid lower eyelid and the reference electrode a few cm laterally to the outer canthus. Signals were amplified and digitized using a sampling rate of 50 kHz, downsampled to 3.3 kHz, and stored for off-line analysis.

### Experimental design

**Figure 1:**
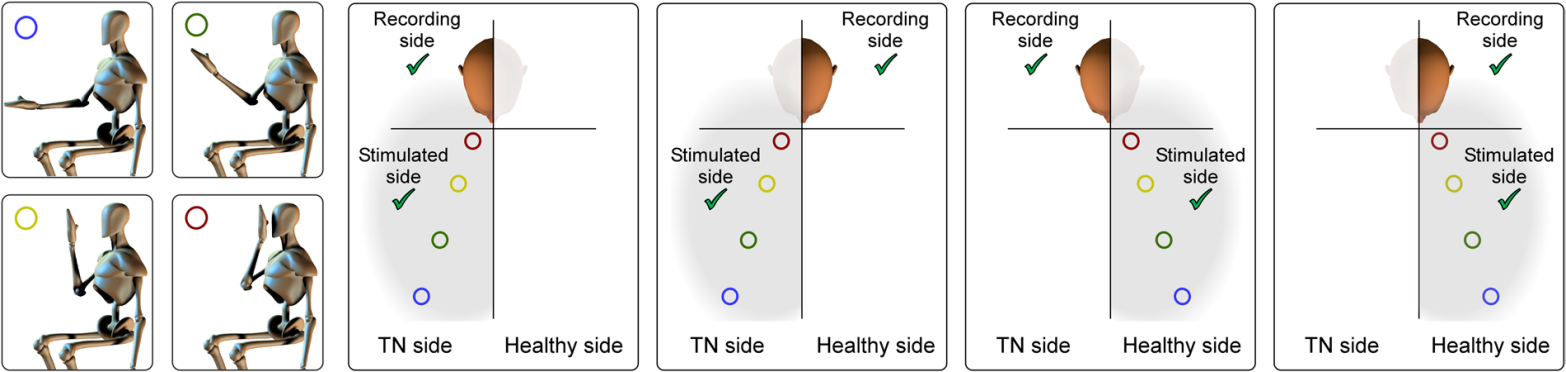
Stimulation and recording conditions. Somatosensory stimuli were delivered to the median nerve at the wrist, while the hand was in four different positions: ‘ultra-far’, ‘far’, ‘near’, and ‘ultra-near’ (first column). Stimuli were delivered to the hand ipsilateral to the TN side (second and third columns), or to the hand contralateral to the TN side (fourth and fifth columns). The hand-blink reflex (HBR) was recorded using surface EMG from the eye ipsilateral to the stimulated hand (second and fifth columns) and from the eye contralateral to the stimulated hand (third and fourth column). This 4 (positions) × 2 (stimulation sides) × 2 (recording sides) design resulted in 16 HBR average responses for each patient.

HBR responses were recorded while the patient held their stimulated hand in four positions, corresponding to the following distances from their face: ultra-far ;60 cm (forearm ~120 degrees relative to arm), far ;40 cm, near ;20 cm, and ultra-near ;4 cm (forearm ~ 75 degrees relative to arm, hand in front of their ipsilateral eye) (Fig. 1). The far and near positions were determined for each patient by measuring the distance between their stimulated hand and the face, and were clearly marked on a panel placed on the side of the patient’s stimulated hand. The experiment consisted of four blocks. In each block, electrical stimuli were delivered to either the right or the left wrist (i.e. the wrist of the arm undergoing the postural manipulation). The order of blocks was balanced across patients. In each block, four electrical stimuli were delivered for each of the four hand positions, for a total of 16 stimuli per block. The order of hand positions called by the experimenter in each block was pseudo-random, with no more than two consecutive stimuli being delivered in the same position. White noise was played throughout the experiment to mask any subtle sound made by the electrical stimulator.

*Preliminary recordings*. The intensity of electrical stimuli was adjusted in each patient by gradually increasing the stimulus intensity until either a clear HBR was observed in three consecutive trials, or the patient declined a further increase^5,19^. Only patients with a clear and stable HBR (i.e. responders) underwent further testing. In previous studies using the exact same experimental procedures in healthy participants, the percentage of responders has been found to be approximately 60%^5,13,19,20,22^.

In the patients with a stable HBR, stimulus intensity ranged between 14 and 80 mA (M = 43.7, SD = 18.2).

### Statistical analysis

EMG signals were analyzed using Letswave (http://nocions.org)^23^. EMG signals from each patient were high-pass filtered (55 Hz) and full-wave rectified. HBR magnitude was calculated as the response area-under-the-curve (AUC) of the rectified EMG^5,13^, and transformed to z-scores within subject. AUCs were averaged separately for each experimental condition, resulting in 16 HBR average magnitudes for each patient. 

### HBR magnitude analysis

To investigate the combined effects of hand position, stimulated hand, and side of face on the z-scored HBR magnitude (AUC), we performed a three-way, repeated-measures analysis of variance (rm-ANOVA; n = 20) with ‘hand position’ (four levels: ultra-far, far, near, ultra-near), ‘stimulated hand’ (two levels: ipsilateral *to TN side*, contralateral *to TN side*), and ‘recorded eye’ (two levels: ipsilateral *to stimulated hand*, contralateral *to stimulated hand*) as within-subjects factors. Greenhouse-Geisser corrections were applied whenever the assumption of sphericity was violated.

### Geometric modelling

**Figure 2:**
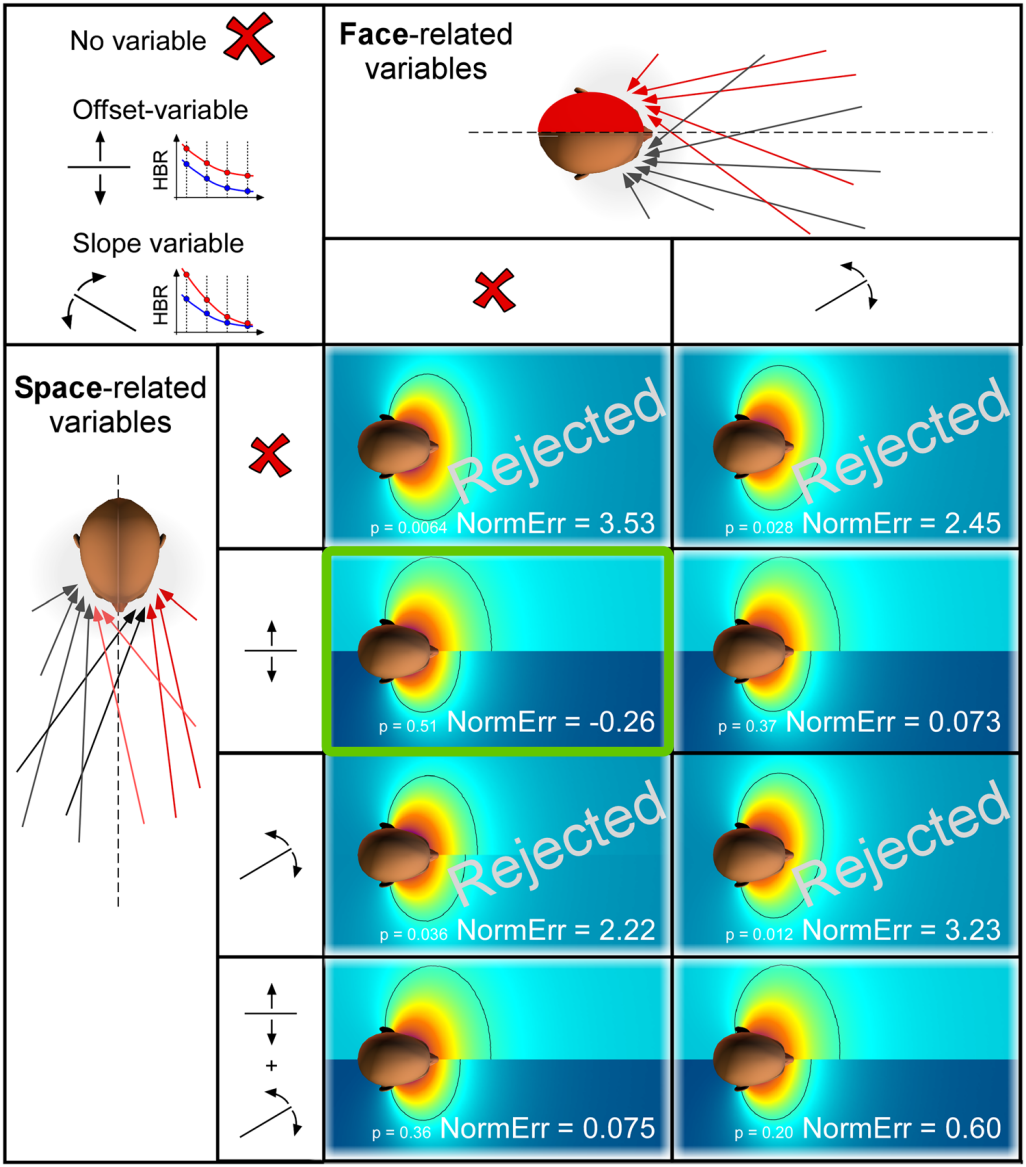
Geometric properties of the DPPS of the face predicted by each of the 16 tested models. We considered two classes of variables, representing underlying neurophysiological explanations for all possible TN-induced changes in DPPS shape and size. The first class of variables reflects that an event *hitting* the side of the *face* affected by TN is estimated to do more harm (columns). The second class of variables reflects that an event *occurring* in the side of *space* ipsilateral to the TN is estimated to do more harm (rows). Since the geometrical modelling calculates the probability of an environmental stimulus to hit the face, and assumes a linear relationship between that hit probability and the HBR magnitude^12^, the first (*face*) explanation could be modelled with one parameter: a change in slope (i.e. a multiplicative factor). In contrast, the second (*space*) explanation could be modelled using up to two variables: either a change in offset (i.e. an additive factor), or a change in slope (i.e. a multiplicative factor), or both. The resulting matrix shows the best model fit resulting from each of all possible combinations of the variables. Note that in some cases, adding a variable has only a very small effect on the resulting fit, which explains why some model fits look very similar. The ‘NormErr’ is a measure of the goodness of fit: it is a normalised measure of how much more error each model gives in its fit to the data than expected. Therefore, the smaller (or more negative) the NormErr, the better the model fit. The p-values indicate whether a model is rejected (P < 0.05) or not (P > 0.05). DPPS models which did not pass goodness-of-fit testing are rejected, whereas the model that fitted the data best is highlighted in green.

Briefly, we applied a geometrical modelling approach to the HBR data obtained by averaging the response between the two eyes (as in Bufacchi *et al*.^12^ and Bufacchi and Iannetti^21^) (Fig. 2). In addition, we tested whether our results were consistent even when considering the data from each eye separately. For the purpose of geometric modelling analysis, the data must be normally distributed and have equal variance. To satisfy these conditions, we calculated the Z-scores of the power-transformed AUCs, as described in more detail elsewhere^12^.

**Figure 3:**
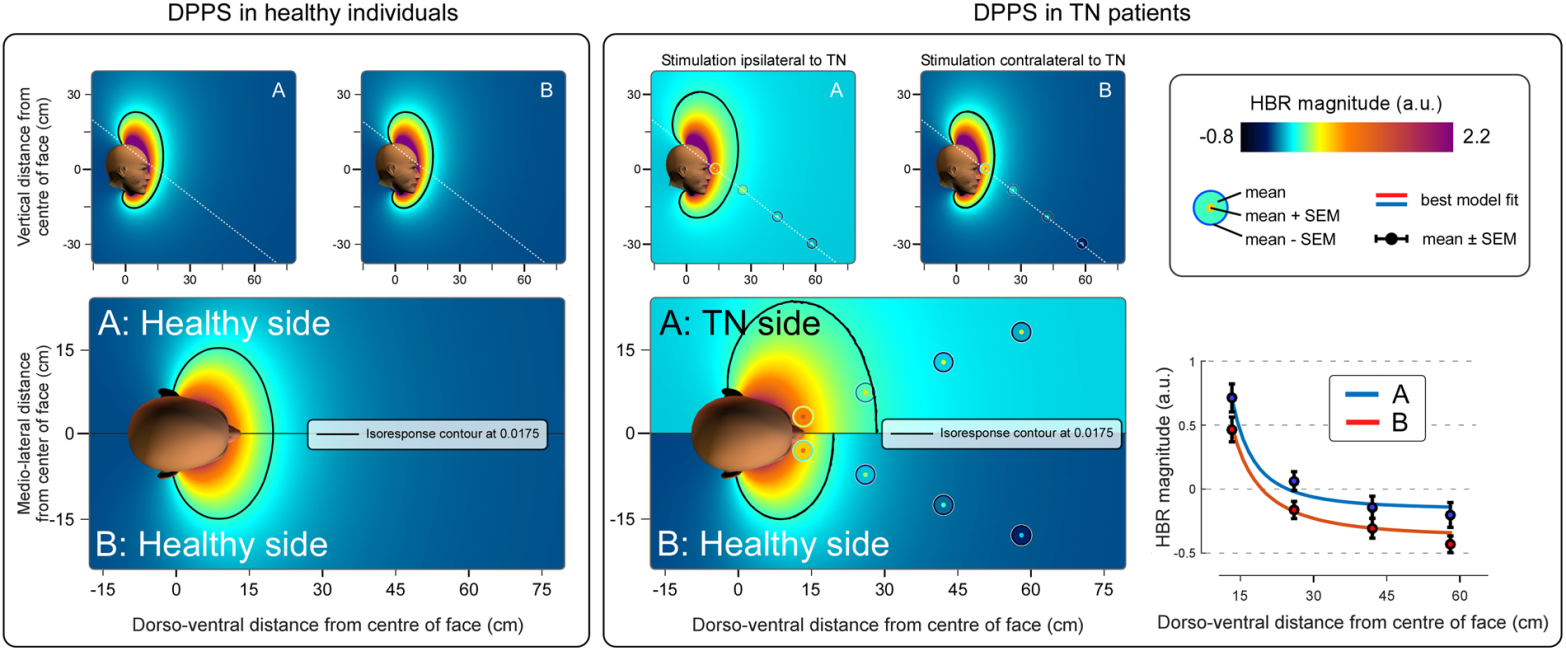
DPPS asymmetry in patients with TN. Colour plots show the geometric properties of DPPS in healthy individuals (left panel, modelling based on the data reported in Bufacchi and Iannetti^21^), and TN patients (right panel) using an axial section passing through both eyes (bottom) and two sagittal sections passing through each eye (top). The dotted line in the sagittal sections indicates the cutting plane used for the axial section. The background colour represents the estimated threat of environmental events predicted by the best-fitting model. The isoresponse contour line indicates the positions at which the predicted HBR magnitude is equal to the average HBR magnitude across all hand positions on the side of space contralateral to the TN (0.0175). The measured, normalised and averaged HBR data are represented as concentric circles located where the measurements were taken. The line graphs at the bottom show the mean HBR magnitude (±SEM) measured at each hand position, together with the HBR magnitude predicted by the best fitting geometric model (coloured lines). The best fitting model indicates that environmental events located in the side of space ipsilateral to the TN are considered to have a higher probability to do harm than events located in the side of space contralateral to the TN. This is reflected in a DPPS asymmetric along the mediolateral axis: the 'defensive bubble' is extended further away from the face on the side of space ipsilateral to the TN.

**Figure 4:**
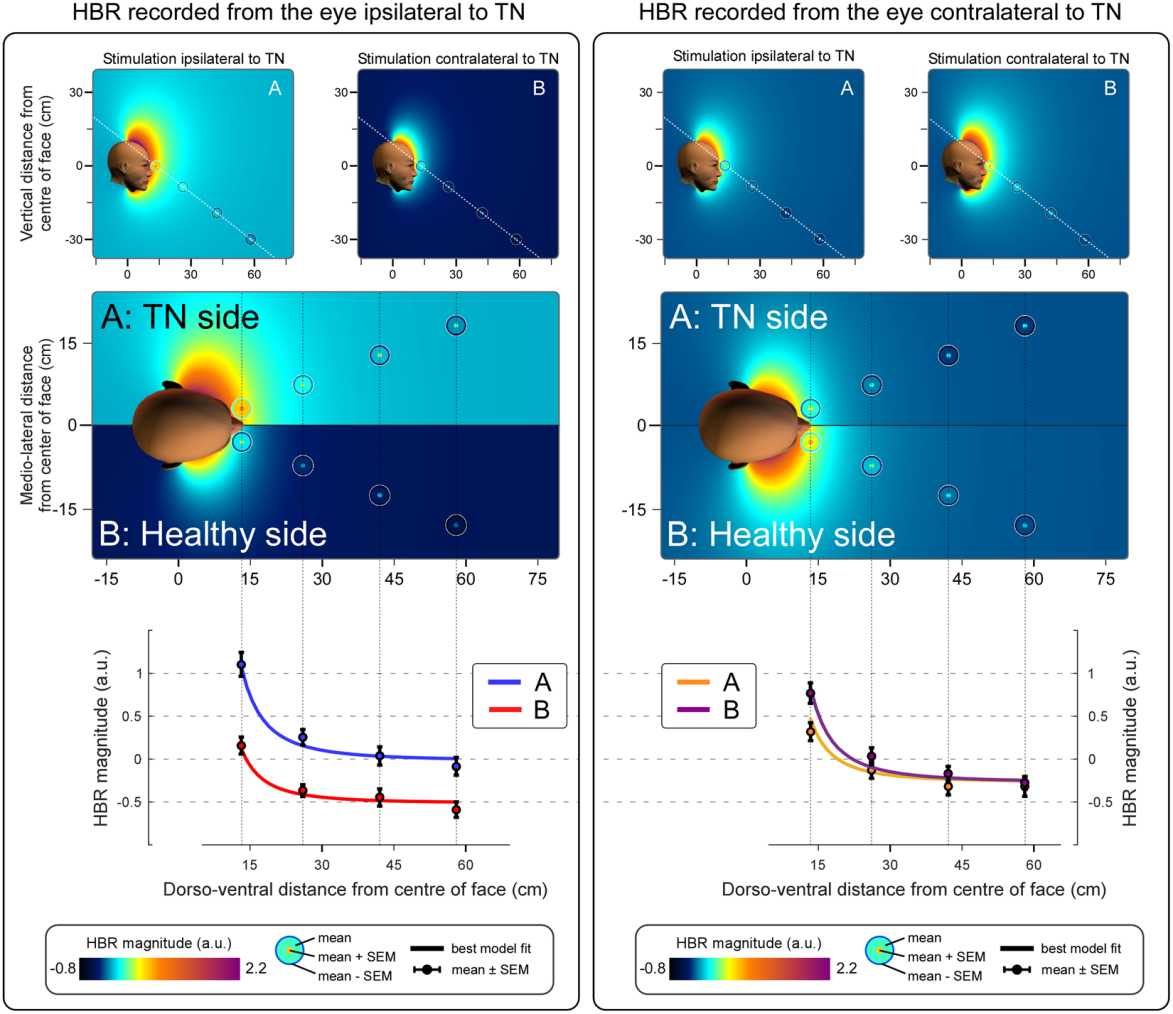
DPPS asymmetry for each separate eye in patients with TN. Colour plots represent the estimated threat of environmental events predicted by the best-fitting model, as estimated from the HBR recorded from *a given eye*. The layout is as described for Fig. 3. Note that in the left panel, the HBR magnitude recorded from the eye contralateral to the TN side is overall lower than the HBR for either side on the right panel. The reason is the so-called ‘ipsi-contra effect’: in healthy participants, the HBR is stronger when recorded from the eye ipsilateral to the stimulated hand^5^. This figure shows the individual responses from each eye, and therefore in the left panel the response for side B (showing the HBR from the eye contralateral to the stimulated hand) is expected to be smaller than for side A (showing the HBR from in the same eye, which in this condition is ipsilateral to the stimulated hand). The fact that in the right panel there is no inverted pattern (i.e. a response weaker on side A – contralateral to the stimulated hand, than on side B – ipsilateral to the stimulated hand), follows directly from the fact that we are dealing with TN patients: in contrast to healthy subjects, patients have a baseline increase of HBR magnitude when stimuli are delivered on the side of space ipsilateral to the TN (side A), resulting in an increase of HBR magnitude on that side.

We considered two possible underlying neurophysiological mechanisms for the changes in DPPS shape and size, which are depicted by the arrows in Fig. 2. The first (*face prioritisation mechanism*) is that the neural system underlying the modulation of the HBR circuitry takes into consideration the increased negative consequence that an external event would cause when hitting the side of the *face* affected by TN. The second (*space prioritisation mechanism*) is that the same system simply assumes that sensory events occurring in the side of peripersonal *space* ipsilateral to the side of the TN represent a higher threat. Because the geometrical model assumes a linear relationship between hit probability and HBR magnitude^12^, the *space prioritisation mechanism* could be modelled using up to two parameters (represented by the space-related variables in the left part of Fig. 2): either a change in slope (i.e. a multiplicative factor), or a change in offset (i.e. an additive factor), or both. This mechanism is exemplified by the red arrows that always originate in the side of *space* ipsilateral to TN regardless of the side of the face they hit. The arrows that hit the TN-affected side of the face are drawn in a lighter colour (note that the exact number of arrows is irrelevant for the analysis). Note also that because we are not modelling how the brain represents the parts of space ipsilateral or contralateral to each arm, we have assumed a step-boundary between the halves of space: the area to the one side of the face’s midline is one side of space, and the area to the other side of the midline is the other side of space. While the sharp boundary depicted in Figs 2–4 is unlikely to be physiologically plausible, the aim of this model was not to characterise the boundary between right and left sides of space, but to distinguish whether the two sides of space have different properties with respect to the DPPS. In contrast to the space prioritisation mechanism, the *face prioritisation mechanism* (represented by the face-related variables in the top part of Fig. 2) could only be modelled using a change in slope (i.e. a multiplicative factor), because the increase in threat in this mechanism is inherently linked to the probability of a threat hitting the side of the face affected by TN. This mechanism is exemplified in the top panel of Fig. 2, by the red arrows always hitting the side of the *face* ipsilateral to TN regardless of the side of space they originate in. We tested all possible combinations of parameters and underlying causes discussed above, resulting in 8 different model variants, each corresponding to another cause of the differences in HBR magnitude observed between the sides of space ipsilateral and contralateral to TN. We used Chi-squared goodness of fit testing to assess which of these models (and corresponding underlying assumptions) did not explain enough variance of the data in order to be accepted^12^. This method also takes into account an important factor – the *parsimoniousness* of the models – by providing a better goodness of fit only if the fit is improved more than expected by adding just any additional variable. Therefore, this method allows us to compare the accepted models to see which one explains most of the variance of the data most effectively.

### Data availability

Anonimised data is freely available upon request.

## Results

### Clinical features of Trigeminal Neuralgia

In 13 patients the TN was on the right side of the face, and in the remaining 7 it was on the left side. The trigeminal divisions affected by TN were as follows: V1 (2 patients), V2 (2 patients), V3 (5 patients), V2-V3 (3 patients), and V1-V2-V3 (8 patients). The duration of pain ranged between 3 months and 30 years (mean = 9.3, SD = 7.3 years).

Interestingly, the type of trigger had some predictive value with respect to the likelihood that an individual was a HBR responder: out of the 7 patients with only intraoral triggers, two were HBR responders (29%). In contrast, out of the 22 patients with extraoral triggers, 18 were HBR responders (82%). These percentages of HBR figures are in stark contrast with the average ~60% of HBR responders that we repeatedly observed in the normal population^5,6,12,13,21,22^. This qualitative observation suggests that when the TN pain is only triggered by sensory events occurring within the mouth, external events are given less behavioural relevance.

### HBR magnitude analysis

The three-way repeated-measures ANOVA showed main effects of ‘hand position’ (F = 43.76, p = 1.15*10^−10^, η^2^ = 0.317), ‘stimulated hand’ (F = 5.533, p = 0.029, η^2^ = 0.034), and ‘recorded eye’ (F = 160.35, p = 1.04*10^−10^, η^2^ = 0.118), as well as a two-way interaction between ‘hand position’ and ‘recorded eye’ (F = 40.87, p = 2.12*10^−10^, η^2^ = 0.039), and a three-way interaction between ‘hand position’, ‘recorded eye’ and ‘stimulated hand’ (F = 3.40, p = 0.044, η^2^ = 0.0032). The main effect of ‘hand position’ indicates that when the hand is close to the face the blink magnitude is increased, as already observed repeatedly in healthy participants^5,6,12,13,21^. The main effect of ‘recorded eye’ confirms the previously-described “ipsi-contra effect”: the HBR is stronger in the eye ipsilateral to the stimulated hand. This effect has also been repeatedly observed in healthy participants^5^. Importantly, the main effect of ‘stimulated hand’ indicates that stimuli delivered on the side of space ipsilateral to the TN elicited an overall stronger HBR (Figs 3 and 4). The ‘stimulated hand’ x ‘hand position’ interaction indicates that the ipsi-contra effect is stronger when the stimulated hand is nearer to the face. Finally, the ‘hand position’ x ‘recorded eye’ x ‘stimulated hand’ interaction indicates that this difference in ipsi-contra effect is larger when the hand ipsilateral to the TN is stimulated than when the contralateral hand is stimulated.

### Geometric modelling

Geometric modelling showed that, in contrast to what was observed in healthy participants^21^, the DPPS of TN patients is clearly asymmetric along the medio-lateral axis, being further extended on the side of space ipsilateral to the TN (Fig. 3). The Goodness of Fit and p-values for each tested model are shown in Fig. 2. The only difference between the models best fitting the HBR modulation in healthy participants and in TN patients consisted of a parameter that modelled the increased threat of stimuli occurring in the side of space ipsilateral to the TN as a change in offset. In other words this model included an additive increase of HBR magnitude when stimuli were delivered on the hand ipsilateral to the TN, regardless of where that hand was positioned. All models that did not include any factor representing an increase of threat on the side of space ipsilateral to TN (either as a multiplicative or as an additive factor) were rejected (Fig. 2).

Therefore, to successfully explain the data it is necessary to assume that the brain considers stimuli occurring within the side of space ipsilateral to TN more dangerous than those occurring on the other side of space. Furthermore, this increase in danger on the affected side of space is likely to be a simple baseline-increase of the threat value of stimuli, rather than a multiplicative effect. Finally, a multiplicative effect of the increased damage done to the affected side of the face does not invalidate the model, but it is not necessary to explain the data.

The additional modelling of the HBR data separately for each eye (Fig. 4) followed exactly the same rules and indicated that even when the eyes are considered individually, it is necessary to assume that the brain deems stimuli occurring within the space ipsilateral to TN more dangerous than those occurring on the other side of space. In Fig. 4 the response displayed on side B of the left panel (i.e. the HBR recorded from the eye ipsilateral to TN, when the hand contralateral to TN is stimulated) is much weaker than the HBR shown anywhere else in that figure, or in Fig. 3. This is consequent to the ‘ipsi-contra effect’, wherein the HBR is stronger for the eye ipsilateral to the stimulated hand^5^. This effect is still present in TN patients, as can be seen by separately comparing sides A and B *between* the left and right panels. However when this effect is combined with the overall stronger response to stimuli delivered to the hand located in the TN side of space, the result is that the HBR response is weakest when (1) recorded from the eye contralateral to the stimulated hand, and (2) elicited contralaterally to TN, which is the case for side B of the left panel of Fig. 4.

## Discussion

In this study we characterized the possible asymmetries in shape and size of the defensive peripersonal space (DPPS) of the face in patients affected by trigeminal neuralgia (TN). In contrast to most other chronic pain conditions, in typical TN pain is rarely spontaneous. In stead in TN, electric-shock-like pain paroxysms are triggered by innocuous stimuli (e.g. a gentle touch or a puff of air) in the affected orofacial territory, which patients soon learn to avoid^24^. Clinically, TN patients become more alert and take a defensive posture when the examining physician approaches their face. Hence we expected that these patients had developed a DPPS with characteristics peculiar to this unique condition. We used a validated method based on the modulation of the hand-blink reflex (HBR) elicited by somatosensory stimulation of the hand located at four different distances from the face^13^. We obtained two main findings. First, stimuli delivered on the side of space ipsilateral to the TN elicit a stronger HBR. Second, the normal ‘ipsi-contra effect’, wherein the HBR is larger for the eye ipsilateral to the stimulated hand^5^, is altered in TN patients. These results indicate that the estimated potential for harm of sensory events is enhanced when they occur in the side of space ipsilateral to the TN – therefore defensive responses are upregulated to reduce the likelihood that external events evoke the painful paroxysms typical of TN.

Geometrical modeling of the HBR enhancement extended these findings, by allowing us to derive the fine-grained spatial properties of the DPPS. In healthy participants the DPPS is anchored to the face and has the shape of a bubble elongated along the vertical axis, and within this bubble HBR responses are enhanced with proximity of the threat to the face, thus defining a high-risk area^12,13^.

In TN patients the HBR magnitude increased monotonically and non-linearly with hand-face proximity (Fig. 3) – a finding repeatedly described in healthy individuals^12,13,21^. Given that (1) we delivered stimuli to both the hand ipsilateral and contralateral to the TN side, and (2) we recorded the HBR from both eyes, we were able to assess possible side asymmetries in DDPS shape. We observed that (1) stimuli delivered on the side of space ipsilateral to the TN elicit an *overall* stronger HBR (Figs 2 and 3), and (2) that the ipsi-contra effect is stronger when the stimulated hand is nearer to the face, and particularly so when it is ipsilateral to the TN (Fig. 4). This is different from what observed in healthy participants, in whom the strength of the ipsi-contra effect does not depend on hand position^5^. Intuitively, these observations suggest that the nervous system estimates sensory events occurring in the peripersonal space ipsilateral to the TN side to be more threatening.

There are at least two possible neural mechanisms underlying this concept. One possibility (*space prioritisation mechanism*) is that the nervous system simply deems environmental stimuli to be more threatening when they occur on the side of *space* ipsilateral to the TN, regardless of the probability that the affected or unaffected side of the face will be hit. This mechanism is exemplified in the left panel of Fig. 2, by the red arrows that always originate in the side of *space* ipsilateral to TN regardless of the side of the face they hit. The other possibility (*face prioritisation mechanism*) is that the nervous system continuously estimates the probability that environmental stimuli will hit either side of the *face*, and assigns a higher potential for harm to hitting events on the affected side of the face, regardless of where these sensory events are located within the peripersonal space. This mechanism is exemplified in the top panel of Fig. 2, by the red arrows always hitting the side of the *face* ipsilateral to TN regardless of the side of space they originate in. Geometric modelling of DPPS allows for mathematical assessment of these options (see Bufacchi *et al*.^12^), and we used this tool to statistically identify the mechanism that best underlies the experimental observations. A model corresponding to the space prioritisation mechanism clearly outperformed the other models (Fig. 2). This winning model entailed an *additive* baseline increase in HBR magnitude only in the side of space ipsilateral to the TN, without any *multiplicative* change in the hand-face proximity effect compared to the unaffected side (Fig. 3). Thus, the defensive bubble of TN patients is asymmetric: on the side of space ipsilateral to the TN, defensive responses show an overall increase in magnitude. In other words, the position of the threat at which the defensive response has a given magnitude is always located further away from the face on the side of the TN (Fig. 3). This has a clear advantage to minimise harm: indeed, threatening stimuli occurring on the side of space ipsilateral to the TN pose a particularly great risk of evoking a pain attack, and thus require more effective defensive motor responses. It is interesting to note that despite the overall differences in HBR magnitude between equal hand positions in the healthy and the affected side, the increase of HBR magnitude between the nearest and furthest position was not different. This suggests that TN changes defensive mechanisms following an additive mechanism, likely mediated by the addition of post-synaptic potentials (whether excitatory or inhibitory) directly on brainstem HBR circuitry, and not by a multiplicative pre-synaptic effect on some other modulatory mechanisms^25^.

A number of chronic pain conditions entail a disrupted cortical representation of the body and the surrounding space^26^. For example, our observation that TN patients assign a particular threat value to one hemispace is reminiscent of the findings in patients affected by unilateral CRPS of the hand^27^. In these patients, the affected hand became less painful when located in the side of space contralateral to its usual location. A possible speculation that can relate these previous observations with what we report here is that in TN patients the brain soon learns to be wary of specific external events and may come to attribute the source of the pain to these external events rather than attributing it to the body.

## Conclusion

These observations demonstrate that the neural mechanisms underlying body protection are altered in patients affected by TN. In these patients, the nervous system purposefully adjusts the estimated potential for harm of sensory events occurring in the side of space ipsilateral to the TN. This adjustment enhances defensive behavior and thereby reduces the likelihood that external events evoke the painful paroxysms typical of this condition.
